# Baicalein Selectively Induces Apoptosis in Activated Lymphocytes and Ameliorates Concanavalin A-Induced Hepatitis in Mice

**DOI:** 10.1371/journal.pone.0069592

**Published:** 2013-07-22

**Authors:** Yan Zhang, Lei Shan, Yaping Hua, Dan Wang, Huawu Zeng, Runhui Liu, Weidong Zhang, Zhenlin Hu

**Affiliations:** 1 School of Pharmacy, Second Military Medical University, Shanghai, China; 2 School of Pharmacy, Shanghai JiaoTong University, Shanghai, China; NIAID, United States of America

## Abstract

**Background:**

Insufficient apoptosis in activated lymphocytes contributes to the development of autoimmune hepatitis (AIH). Baicalein (BE), a flavonoid originally isolated from the root of *Scutellaria baicalensis* Georgi, possesses anti-inflammatory properties. However, whether BE can selectively induce apoptosis in activated lymphocytes and exert therapeutic effect on AIH has not been studied.

**Methodology/Principal Findings:**

The pro-apoptotic properties of BE were evaluated *in vitro* on different types of immune cells, and *in vivo* effects of BE were examined in a murine model of Concanavalin A (Con A)-induced hepatitis. *In vitro* treatment with BE resulted in a higher increase in the level of apoptosis in Con A-stimulated murine splenocytes, Con A-stimulated CD3^+^ splenocytes, lipopolysaccharide (LPS)-stimulated CD19^+^ splenocytes, and phorbol 12-myristate 13-acetate/ionomycin-stimulated Jurkat T cells, compared with that in unstimulated naïve ones. Murine bone marrow-derived dentritic cells, peritoneal macrophages, and RAW264.7 cells, either stimulated with LPS or unstimulated, were all insensitive to the BE-induced apoptosis. BE treatment also led to a loss of mitochondrial membrane potential, an increase of cytochrome c release from mitochondria to the cytosol, a decrease in the ratio of Bcl-2/Bax, and activation of caspase-9,-3 in Con A-stimulated CD3^+^ splenocytes and LPS-stimulated CD19^+^ splenocytes, while showing no impact on Fas/FasL expressions and caspase-8 activation. *In vivo* administration of BE alleviated Con A-induced liver injury, suppressed serum level of TNF-α and IFN-γ, and reduced liver infiltration of mononuclear cells (MNCs). Furthermore, BE treatment increased the incidences of apoptosis in liver-infiltrating MNCs and splenocytes, as well as in CD3^+^ and CD19^+^ splenocytes. When liver MNCs and splenocytes from BE-treated mice were cultured *in vitro* for 24 h, they exhibited marked increase in apoptosis compared to vehicle-treated control.

**Conclusions/Significance:**

The present study demonstrates the ability of BE to promote apoptosis in activated lymphocytes through mitochondrial pathway and its potential use in the treatment of AIH.

## Introduction

Autoimmune hepatitis (AIH) is a disease characterized by progressive liver inflammation of unknown etiology that may advance to fibrosis and cirrhosis [1]. The pathogenic mechanisms of AIH still remain unclear. The liver inflammation in AIH encompasses both cell-mediated cytotoxicity by infiltrating lymphocytes and the production of autoantibodies. Therefore, abnormality in immune regulation is thought to be implicated in the pathogenesis of this disease [2]. Currently, the only viable treatments of AIH are immunosuppressant application and liver transplantation. But long term applications of currently available immunosuppressive drugs carries serious risks [3]. Therefore, it is significantly important to develop new specific drugs.

Deletion of activated and autoreactive lymphocytes by apoptosis is a critical mechanism by which the immune system maintains homeostasis [4], [5]. There is increasing evidence that abnormalities in this process might contribute to the development of AIH. It has been reported that the activated lymphocytes in patients with AIH fail to down-regulate the expression of the antiapoptotic protein, bcl-2, which may protect them from apoptosis and thereby extend the disease process [6]. Fas/Fas-ligand system is known to plays a key role in the control of activation-induced apoptosis of lymphocytes [7]. Four polymorphisms of human Fas gene have been associated with the occurrence of AIH in Japan [8], and one polymorphism in the Fas gene promoter at position −670 has been associated with the early development of cirrhosis in white North American and Northern European patients with AIH [9]. These data suggests that genetic factors that affect the integrity of the apoptotic pathway can influence the progression of AIH possibly by perpetuating the survival of activated lymphocytes. Concanavalin A (Con A)-induced hepatitis is considered to be an experimental murine model of AIH [10], [11]. A previous study demonstrated that CD44-knockout mice exhibited enhanced pathogenesis in Con A-induced hepatitis, primarily due to inability of Con A-activated CD44-deficient T cells to undergo apoptosis [11]. A more recent study demonstrated that Galectin-3 deficiency prevents Con A-induced hepatitis partly due to enhanced apoptosis in both liver-infiltrated mononuclear cells (MNCs) and splenocytes [12]. These evidence further support the notion that dysregulation of apoptosis in activated lymphocytes is involved in the pathogenesis of AIH.

Since insufficient apoptosis in activated lymphocytes is an essential factor contributing to the development of AIH, specific induction of apoptosis in activated lymphocyte should be beneficial in depressing excess immune responses and inducing long-lasting immunological tolerance, and thus represents a new therapeutic strategy for AIH. Recently, a few compounds have been reported to selectively induce or potentiate apoptosis in activated T cells and inhibit the development of autoimmune diseases in several animal models, including experimental allergic encephalitis [13], [14], adjuvant arthritis [13], and Con A-induced hepatitis [15]. These studies further demonstrated the feasibility of treatment of autoimmune diseases by agents that induce apoptosis of activated T cells.

Natural compounds purified from herbal medicines that have known indications for inflammatory disease conditions often have a low toxicity profile [16], [17]. Therefore, exploring new immunosuppressive agents that selectively induce apoptosis in activated lymphocytes from herbal medicines is conducive to the development of novel effective pharmaceuticals for AIH. Baicalein (BE) is a flavonoid originally isolated from the root of *Scutellaria baicalensis* Georgi, known as “Huang qin” in China [18]. This herb has been widely used in treatment of various diseases such as hepatitis, pneumonia, and diarrhea [19]. Previous studies have demonstrated that BE possesses potent anti-inflammatory properties [20]–[25]. However, the underlying mechanism has not been fully elucidated. Of note, BE has been reported to induce apoptosis in several cancer cell lines, including human pancreatic [26], breast [27], prostate [28], and gastric [29] cancer cells. The pro-apoptotic effects of BE on both lymphocytes cell line and human peripheral blood mononuclear cells has also been shown previously [30], [31]. However, whether it can selectively induce apoptosis in activated lymphocytes has not been studied. In present study, we at first conducted a series of *in vitro* experiments to investigate the pro-apoptotic effect of BE on various immunocytes of different activating status, and then used Con A-induced hepatitic mice as an animal model to determine whether BE could induce lymphocyte apoptosis *in vivo* and has therapeutic potential for AIH. We demonstrated that BE selectively induced apoptosis in activated lymphocytes and ameliorated Con A-induced hepatitis in mice. Our findings indicate that BE may have a therapeutic value in the treatment of AIH and provides a new cellular mechanism for its therapeutic effect.

## Methods

### Ethics Statement

All animal experiments were approved by the Administrative Committee of Experimental Animal Care and Use of Second Military Medical University (SMMU, Licence No. 2011023), and conformed to the National Institute of Health guidelines on the ethical use of animals.

### Reagents

BE (purity >99%) was purchased from National Institute for the Control of Pharmaceutical and Biological Products, China. Con A, lipopolysaccharide (LPS), phorbol 12-myristate 13-acetate (PMA), and ionomycine were purchased from Sigma (St. Louis, MO, USA). PE-conjugated monoclonal antibodies (PE-mAbs) recognizing CD3, CD19, Fas, or FasL and FITC-conjugated mAb against active caspase-3 were purchased from BD PharMingen.

### Mice

C57BL/6 mice (6-week-old, female) were purchased from Shanghai SLAC Laboratory Animal Co., LTD (Shanghai, China). All animals were acclimatized under controlled temperature (20±2°C), humidity (60±5%) and 12 h light/12 h dark cycle for 1 week before the experiment.

### Cell Preparation and Culture

All cells were cultured in RPMI 1640 medium (Sigma) supplemented with 10% fetal bovine serum (Gibco) at 37°C in a 5% CO_2_ atmosphere. Splenocytes were prepared by passing disrupted spleen collected from C57BL/6 mice through a 70-µm nylon cell strainer (BD Labware), and depleting red blood cells by 5-minute incubation with ammonium chloride (0.8% [weight/volume]). CD3^+^ cells and CD19^+^ cells were isolated from splenocytes by magnetic-activated cell separation (MACS)-based purification using Pan T Cell Isolation Kit and B Cell Isolation Kit (Miltenyi Biotec), respectively, according to the manufacturer’s instructions, and the purity of obtain cells was more than 95% as determined by flow cytometry. Bone marrow (BM) cells were prepared by flushing the femurs of C57BL/6 mice, filtering through a 70-µm nylon cell strainer, and depleting red blood cells by 5-minute incubation with ammonium chloride (0.8% [weight/volume]). Dendritic cells (DCs) were generated by culturing BM cells with 10 ng/ml of recombinant mouse granulocyte-macrophage colony stimulating factor and interleukin-4 (both from R&D Systems) for 6 days as previously described [32]. To stimulate DCs maturation, LPS (500 ng/ml) was added to the culture on day 5. Peritoneal macrophages were obtained by plating cells lavaged from the mouse peritoneal cavity in 24-well flat-bottomed plates and removing nonadherent cells after 3 hours of culture [33]. The obtained macrophages were stimulated with LPS (500 ng/ml) for additional 24 h to activated them. Jurkat T cells and Raw264.7 macrophage cells were stimulated for 24 h with PMA (25 ng/mL)/ionomycin (1 µM) and LPS (500 ng/ml), respectively, to induce their activation.

### Apoptosis Assay

Cells were stained with FITC-labeled annexin V (annexin V-FITC) and propidium iodide (PI) (BD PharMingen, San Diego, California, USA) according to the manufacturer’s instructions, and analyzed in a FACS Calibur flow cytometer (BD Biosciences, San Jose, CA, USA). The percentages of apoptotic cells were determined as the sum of annexin V-FITC single-positive and annexin V-FITC/PI double-positive cells. In some experiments, Caspase-3 activation was determined using the FITC Active Caspase-3 Apoptosis kit (BD Pharmingen, San Diego, CA, USA) according to the manufacturer’s instructions. In brief, the cells were fixed and permeabilized by adding 100 µl Cytofix/cytoperm solution at 4°C for 20 min, and the cells were then washed with Perm/Wash solution. The cells were stained intracellularly with FITC-conjugated anti-active caspase-3 antibody for 30 min at room temperature and subsequently analyzed by FACS Calibur Flow Cytometer (BD Biosciences, San Jose, CA, USA).

### Assay for Expression of Fas and FasL

Expression of Fas and FasL on lymphocytes was examined by flow cytometry after staining the cells with PE-conjugated mAb recognizing Fas or FasL (BD Biosciences Pharmingen, San Diego, CA, USA ) according to the manufacturer’s instructions.

### Assay for Mitochondrial Membrane Potential

Loss of mitochondrial membrane potential (ΔΨm) was determined by flow cytometry using J-aggregate forming lipophilic cationic probe JC-1 (Molecular Probes, Eugene, OR, USA) staining following the manufacturer’s protocol.

### Subcellular Fractionation and Western Blotting

The preparation of whole cell lysates and mitochondrial and cytosolic fractions were performed as detailed previously [34]. The protein concentrations were determined by Bradford assay (Bio-Rad, Hercules, CA, USA). The proteins (20 µg) were separated by SDS-PAGE and electrophoretically transferred onto PVDF membrane (Perkin-Elmer, Wellesley, MA, USA). The membranes were probed with anti-cytochrome c, anti-COX IV, anti-α-tubulin, Anti-Bcl-2, anti-Bax, and anti-β-actin antibodies (Santa Cruz Biotechnology, CA, USA) overnight at 4°C. Blots were visualized using IRDye 800CW Goat Anti-Mouse Secondary Antibody (LI-COR Biotechnology, NE, USA). Detection was performed with an Odyssey infrared imaging system (LI-COR Biotechnology, NE, USA).

### Caspase-3,8,9 Colorimetric Assay

The activation of caspase-3,8,9 in the cells was measured with colorimetric assay kits (Sigma) according to the manufacturer’s protocol. The caspase activity was expressed as a fold increase of test samples relative to non-treatment control.

### Con A-induced Hepatitis Model

Hepatic damage was induced by a single intravenous injection of Con A (15 mg/kg) into C57BL/6 mice. BE was administered intraperitoneally (i.p.) (100 mg/kg) immediately after Con A injection. Control mice were given i.p. the same volume of solvent instead of BE. Blood samples were collected from mice at 0, 8, and 24 h after Con A injection. Serum levels of alanine aminotransaminase (ALT) were measured using a colorimetric assay kit (Nanjing Jiancheng Bioengineering Institute, Nanjing, China). Serum levels of IFN-γ, TNF-α, and IL-4 were measured using ELISA kits (R&D Systems) according to manufacturer’s instructions. For histological analysis, livers were collected at 24 h after Con A injection. Liver samples were fixed in 10% formalin solution, embedded in paraffin, sectioned, and stained with hematoxylin-eosin. To detect the *in vivo* apoptosis in lymphocytes, the liver-infiltrating MNCs and splenocytes were isolated 24 h after Con A injection as described previously [35], and stained for different markers of cell subsets (CD3 and CD19) or with FITC-annexin/PI. The stained cells were counted using a BD FACS Calibur flow cytometer. In some experiments, the liver MNCs and splenocytes were harvested at 8 after Con A injection. The cells obtained from each mouse were firstly cultured in 24-well flat-bottomed plates for 2 h to allow macrophage adherence, then the nonadherent cells were collected and further cultured *in vitro* for 12 and 24 h, and the percentages of apoptosis at each time point were determined by flow cytometry using FITC-annexin V/PI staining. After 24 hours of *in vitro* culture, the absolute numbers of apoptotic cells in total nonadherent cells derived from liver MNCs and splenocytes, as well as in CD3^+^ T cell and CD19^+^ B cell subsets, were measured by cell counting and flow cytometry using PE-anti-CD3/FITC-annexin V staining and PE-anti-CD19/FITC-annexin V staining, respectively.

### Statistical Analysis

Data are expressed as the mean ± SEM. Statistical analysis was performed using one-way analysis of variance (ANOVA) test, followed by Dunnett’s multiple comparison post hoc test. P values less than 0.05 were considered significant.

## Results

### BE Selectively Induces Apoptosis in Mitogen-activated Lymphocytes

**Figure 1:**
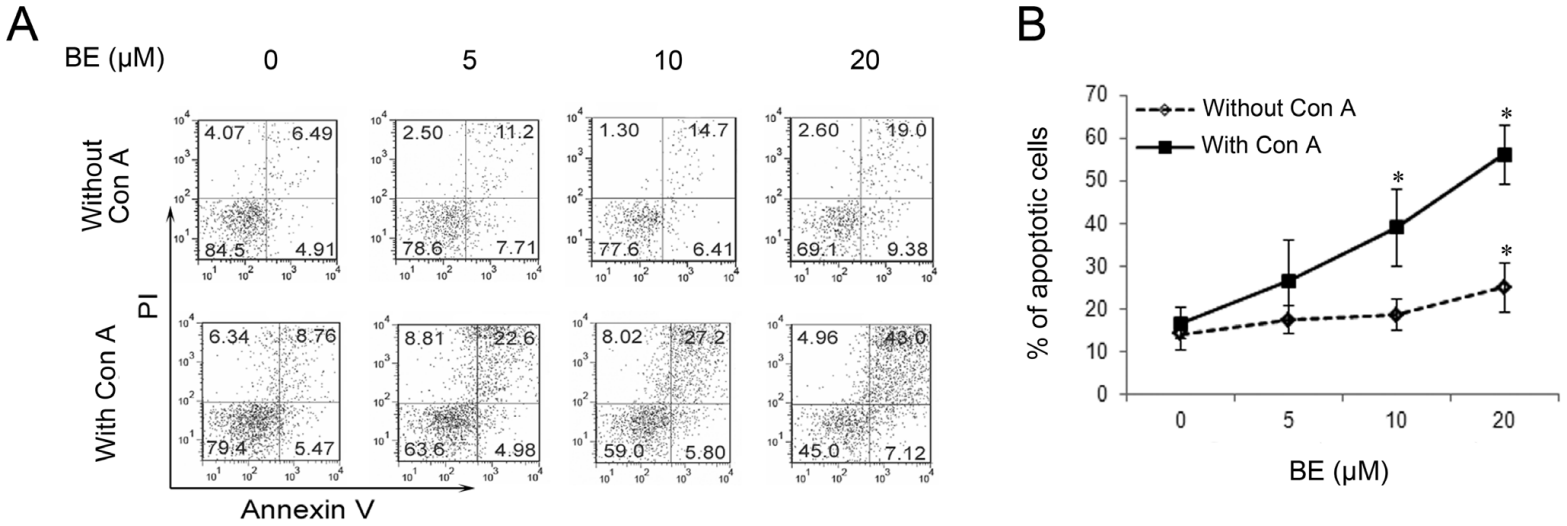
BE preferentially induces apoptosis in mitogen-activated splennocytes. Murine splenocytes were treated with indicated concentrations of BE for 24 h in the absence or presence of 5 µg/ml of Con A, and the percentages of apoptosis were detected using Annexin V/PI staining. (A) is a representative of three independent assays. (B) represents mean ± SEM of three independent experiments. *P<0.05 versus untreated controls.

The pro-apoptotic effect of BE was at first tested on splenocytes. To determine whether BE may exert differential effects on activated and naïve immune cells, freshly isolated murine splenocytes were cultured with BE (5–20 µM) for 24 h in the presence or absence of 5 µg/ml of Con A, and cell death was determined using Annexin V/PI staining. The result revealed an increase in the number of apoptotic events in splenocyte cultures upon BE treatment. Of note, the increase in the levels of apoptosis in splenocyte cultures containing both Con A and BE were markedly higher than that in the corresponding levels detected for the same concentrations of BE when the cells were incubated with BE alone (Fig. 1). This result indicated that BE is more effective in inducing apoptosis in mitogen-activated splenocytes than in naïve ones.

**Figure 2:**
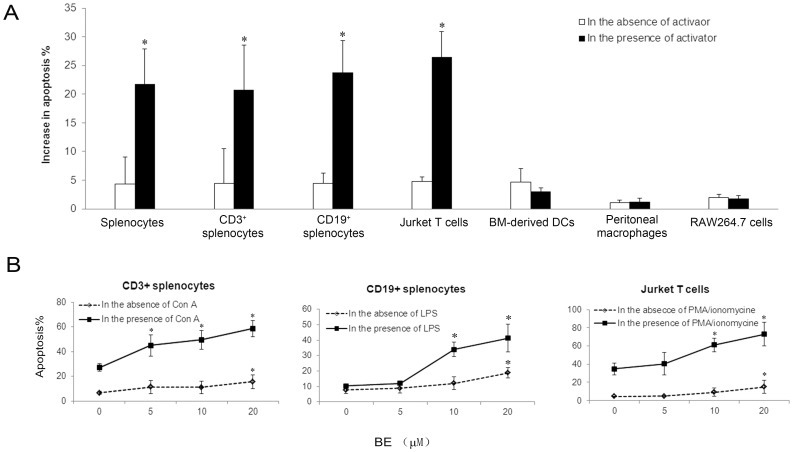
BE selectively induces apoptosis in activated lymphocytes. (A) Different types of immune cells as indicated were incubated with 10 µM of BE for 24 h in the absence or presence of activator, and the percentages of apoptosis were detected using Annexin V/PI staining. The increase in the percentages of apoptosis in each type of cells upon BE exposure was calculated. Following activators were used: 5 µg/ml of Con A for splenocytes and CD3^+^ splenocytes; 500 ng/ml of LPS for CD19^+^ splenocytes, BM-derived DCs, peritoneal macrophages, and RAW264.7 cells; 25 ng/mL of PMA and 1 µM of ionomycin for Jurkat T cells. Data are mean ± SEM of three independent experiments. *P<0.05 versus BE only control. (B) CD3^+^ splenocytes, CD19^+^ splenocytes, and Jurkat T cells were treated with indicated concentrations of BE for 24 h in the absence or presence of respective activator, and the percentages of apoptosis were detected using Annexin V/PI staining. Data are mean ± SEM of three independent experiments. *P<0.05 versus untreated controls.

We next compared the abilities of BE to induced apoptosis in different types of immune cells under different activating status. In this experiment, CD3^+^ cells and CD19^+^ cells were purified from murine splenocytes and were used as T and B lymphocytes, respectively. DCs were derived from murine BM cells and macrophages were obtained from murine peritoneal exudate cells. Besides these primary immune cells, Jurkat T cell line and RAW264.7 cell line were also included in this experiment. For induction of cell activation, splenocytes and T lymphocytes were stimulated with 5 µg/ml of Con A; B lymphocytes, BM-derived DCs, peritoneal macrophages, and RAW264.7 cells were stimulated with 500 ng/ml of LPS; Jurkat T cells were stimulated with 25 ng/mL of PMA plus 1 µM of ionomycin. All of these cells were cultured with 10 µM of BE for 24 h in the presence or absence of the respective activator and cell apoptosis was determined using Annexin V/PI staining. Because the spontaneous apoptosis levels were different among various types of cells, the pro-apoptotic effects of BE were indicated as increase in the percentages of apoptosis in each type of cells upon BE exposure. As shown in Fig. 2A, BE exposure resulted in a significantly higher increase in the level of apoptosis in splenocytes, CD3^+^ splenocytes, CD19^+^ splenocytes, and Jurkat cells in the presence of activator compared with that in the absence of activator. However, BE, either alone or in the presence of LPS, only induced a slight and similar increase of apoptosis in DCs, and induced almost no increase in peritoneal macrophages and RAW264.7 cells. This result suggested BE could selectively induced the apoptosis in activated T and B lymphocytes. We further determine the dose-dependency of BE in inducing apoptosis of lymphocytes by incubating CD3^+^ splenocytes, CD19^+^ splenocytes, or Jurkat cells with increasing concentrations of BE for 24 hours in the absence or presence of the respective activator and subsequently analyzing apoptosis. The results indicated BE enhanced the apoptosis of these tested cells in a dose-dependent manner within the range of 5–20 µM much more profoundly in the presence of activator than in the absence of activator (Fig. 2B). Taken together, above results demonstrated that activated T and B cells are more sensitive to BE-induced apoptosis than naïve ones, whereas DCs and macrophages, either activated or naïve, are insensitive to BE-induced apoptosis.

### BE Induces Apoptosis in Activated Lymphocytes through Mitochondrial Pathway

**Figure 3:**
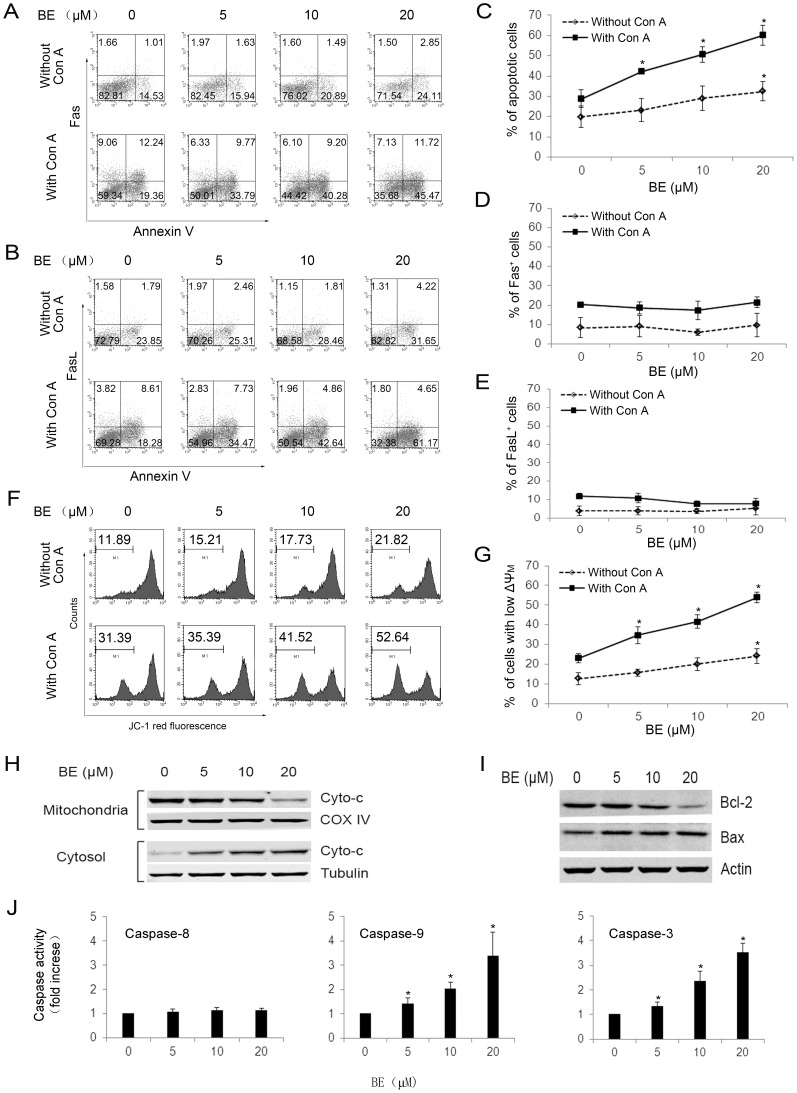
BE selectively induces apoptosis of Con A-activated CD3^+^ T cells through mitochondrial pathway. CD3^+^ T cells were isolated from murine splenocytes using Miltenyi MACS Purification and incubated with indicated concentrations of BE for 24 h in the absence or presence of 5 µg/ml of Con A. (A–E) the percentages of Annexin V^+^, Fas^+^, and FasL^+^ cells were analyzed using PE-anti-Fas mAb/annexin V-FITC or PE-anti-FasL mAb/annexin V-FITC staining. A is a representative of three independent assays with PE-anti-Fas mAb/annexin V-FITC staining. B is a representative of three independent assays with PE-anti-FasL mAb/annexin V-FITC staining. C–E represents mean ± SEM of three independent experiments. (F, G) Loss of ΔΨm in T cells was analyzed using JC-1 staining. F is a representative of three independent assays, and G represents mean ± SEM of three independent experiments. (H) The release of cytochrome c (Cyto-c) from mitochondria in T cells after BE treatment in the presence of Con A was examined by Western blotting. (I) Protein levels of Bcl-2 and Bax in T cells after BE treatment in the presence of Con A were examined by Western blotting. The results shown in H and I are representative of three experiments. (J) The activities of caspase-3, 8, 9 in T cells after BE treatment in the presence of Con A was measured using colorimetric assay. Each column represents the mean ± SEM of 3 experimental values. *P<0.05 versus untreated controls.

In order to dissect possible pathway involved in BE-induced apoptosis in lymphocytes, we next investigated the effects of BE on the expressions of Fas/FasL, loss of ΔΨm, and activation of caspases in immunobead-purified CD3^+^ splenocytes in the absence or presence of 5 µg/ml of Con A. Consistent with the result in Fig. 2B, BE (5–20 µM) markedly increased the percentage of apoptotic (annexin V^+^) cells dose-dependently in the presence of Con A, while only showing slight effects in the absence of Con A (Fig. 3 A–C). At the same time, even though the presence of Con A resulted in a small increase in the expressions of Fas and FasL, they are not further affected by BE treatment (Fig. 3A, B, D, and E). However, BE treatment led to a much more profound loss of ΔΨm in T cells in the presence of Con A than in the absence of Con A (Fig. 3F and G). With the loss of ΔΨm, the release of cytochrome c form mitochondrion to cytosol was markedly increased in T cells upon BE treatment in the presence of Con A (Fig. 3H). In addition, BE treatment also resulted in a down-regulation in the levels of the anti-apoptotic protein, Bcl-2, and an up-regulation in the level of the pro-apoptotic protein, Bax, in T cells in the presence of Con A (Fig. 3I). The colorimetric assay for caspase-3,8,9 further demonstrated that in the presence of Con A, BE treatment led to the activation of caspase-9 and −3 rather than caspase-8 in T cells (Fig. 3J). These results not only confirmed the selectively pro-apoptotic effects of BE on activated T cells but also indicated that BE exerts it effects through mitochondrial pathway rather than death-receptor-mediated pathway.

**Figure 4:**
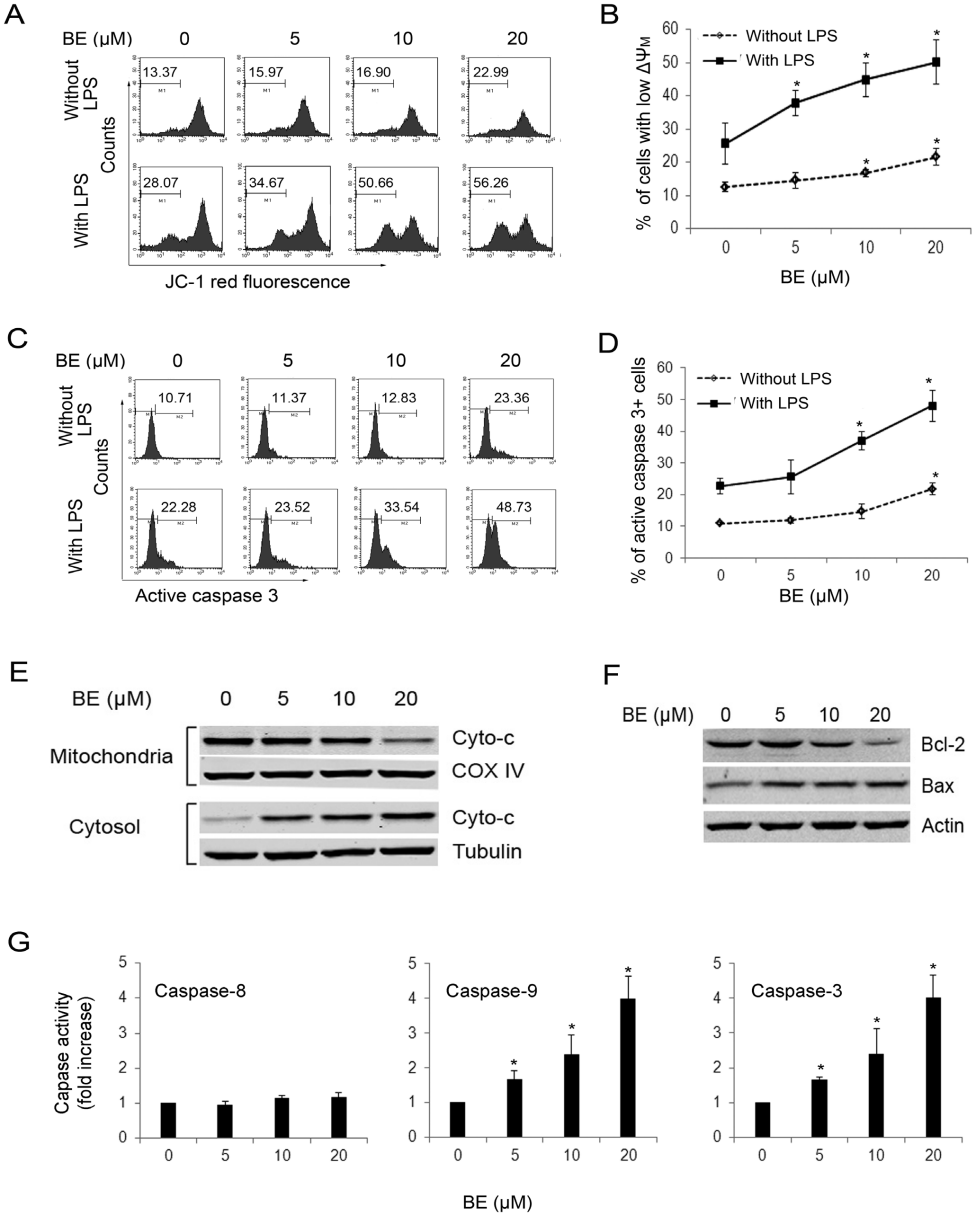
BE selectively induces apoptosis of LPS-activated CD19^+^ B cells through mitochondrial pathway. CD19^+^ B cells were isolated from murine splenocytes using Miltenyi MACS Purification and incubated with indicated concentrations of BE for 24 h in the absence or presence of 10 µg/ml of LPS. (A, B) The percentages of cells with low ΔΨm were analyzed by flow cytometry using JC-1 staining. (C, D) The percentages of active caspase-3+ cells were analyzed by flow cytometry using FITC-anti-active caspase-3 mAb staining. A and C demonstrate representative experiment of three assays. B and D show mean ± SEM of three independent experiments. (E) The release of cytochrome c (Cyto-c) from mitochondria in B cells after BE treatment in the presence of LPS was examined by Western blotting. (F) Protein levels of Bcl-2 and Bax in B cells after BE treatment in the presence of LPS were examined by Western blotting. The results shown in E and F are representative of three experiments. (G) The activities of caspase-3, 8, 9 in B cells after BE treatment in the presence of LPS was measured using colorimetric assay. Each column represents the mean ± SEM of 3 experimental values. *P<0.05 versus untreated controls.

We also tested the expressions of Fas and FasL in immunobead-purified CD19^+^ splenocytes which were treated with increasing concentrations of BE for 24 h in the absence or presence of 10 µg/ml of LPS, and found that they were not influenced by BE treatment (data not shown). However, flow cytometry analysis showed that treatment with BE increased the percentage of cells with low ΔΨm (Fig. 4A and B) and active caspase-3^+^ cells (Fig. 4C and D) in CD19^+^ cells much more profoundly in the presence of LPS than in the absence of LPS. Western blotting assays showed that the release of cytochrome c form mitochondrion to cytosol in B cells after BE treatment in the presence of LPS was marked increased (Fig. 4E). BE treatment also resulted in a down-regulation in the levels of Bcl-2 and an up-regulation in the level of Bax in B cells in the presence of LPS (Fig. 4F). The colorimetric assay for caspase-3,8,9 further demonstrated that in the presence of LPS, BE treatment led to the activation of caspase-9 and −3 rather than caspase-8 in B cells (Fig. 4G). These results further confirmed BE selectively promotes apoptosis of activated B cells through mitochondrial pathway.

### Administration of BE Attenuated Con A-induced Hepatitis and Enhanced Apoptosis of Lymphocytes in Liver-infiltrating MNCs and Splenocytes

**Figure 5:**
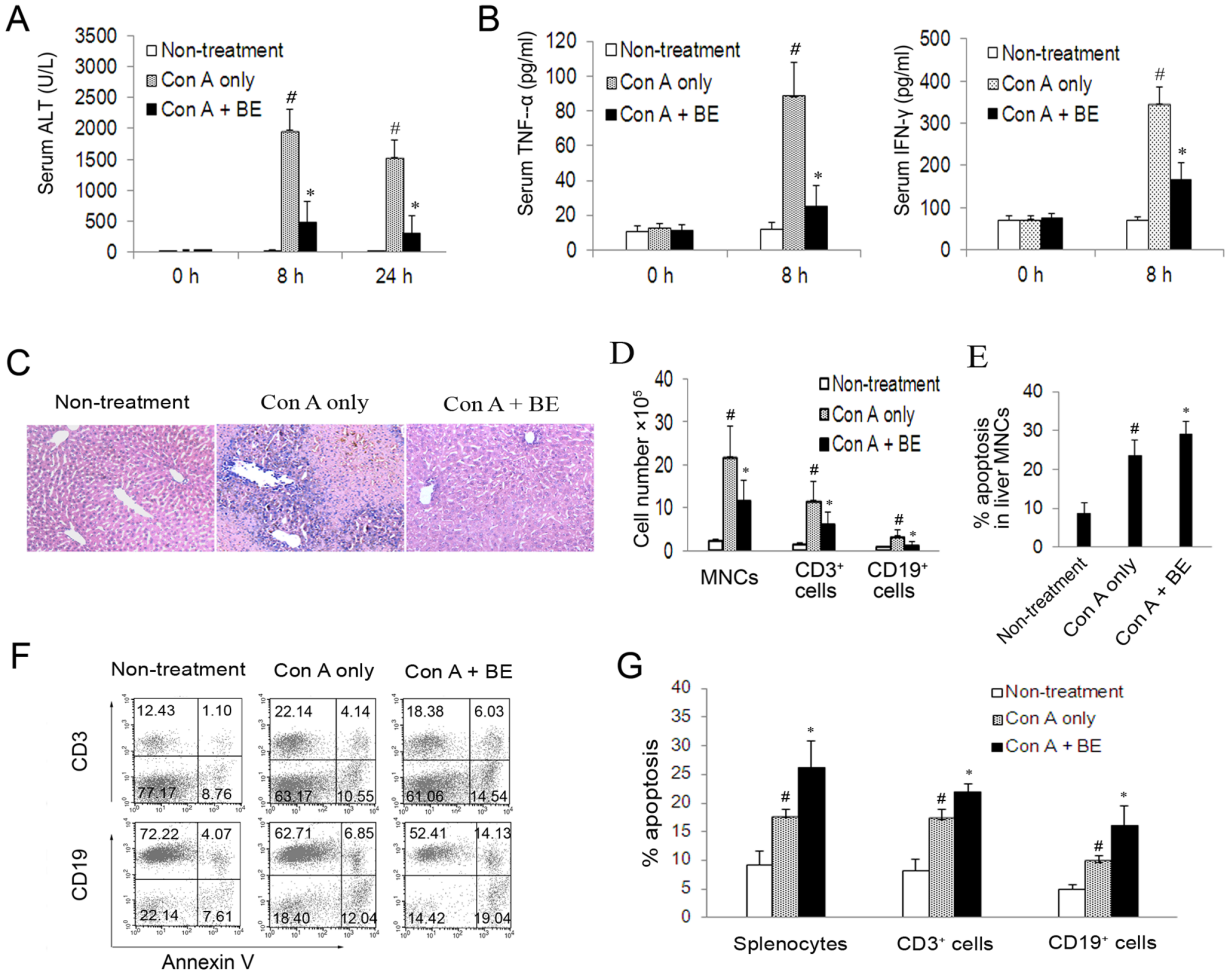
BE protected mice against Con A-induced hepatitis and increased the incidences of apoptosis in liver-infiltrating MNCs and splenocytes, as well as in CD3^+^ and CD19^+^ splenocytes. Murine hepatitis was induced by an intravenous injection of Con A at a dose of 15 mg/kg. BE (100 mg/kg) was administrated intraperitoneally immediately after Con A injection. Blood samples were collected at 0, 8, and 24 h after Con A injection. Livers and spleens were collected 24 h after Con A injection. (A) Serum ALT levels at indicated time points after Con A injection. (B) Serum levels of IFN-γ and TNF-α at indicated time points after Con A injection. (C) Photomicrographs of representative H&E stained liver sections (×200). (D) Cell numbers of MNCs, CD3^+^ T cell and CD19^+^ B cells in livers at 24 h after Con A injection. (E) Percentages of apoptosis in liver MNCs detected by flow cytometry using FITC-annexin V/PI staining. (F and G) Flow cytometry analysis of apoptosis in splenic T and B cells using PE-anti-CD3/staining and PE-anti-CD19/FITC-annexin V staining, respectively. F shows representative results of 10 mice in each experimental group. Data in A, B D, E, and G are mean ± SEM of 10 mice/group. *P<0.05 versus Con A only control, ^#^ *P*<0.05 versus Non-treatment control.

**Figure 6:**
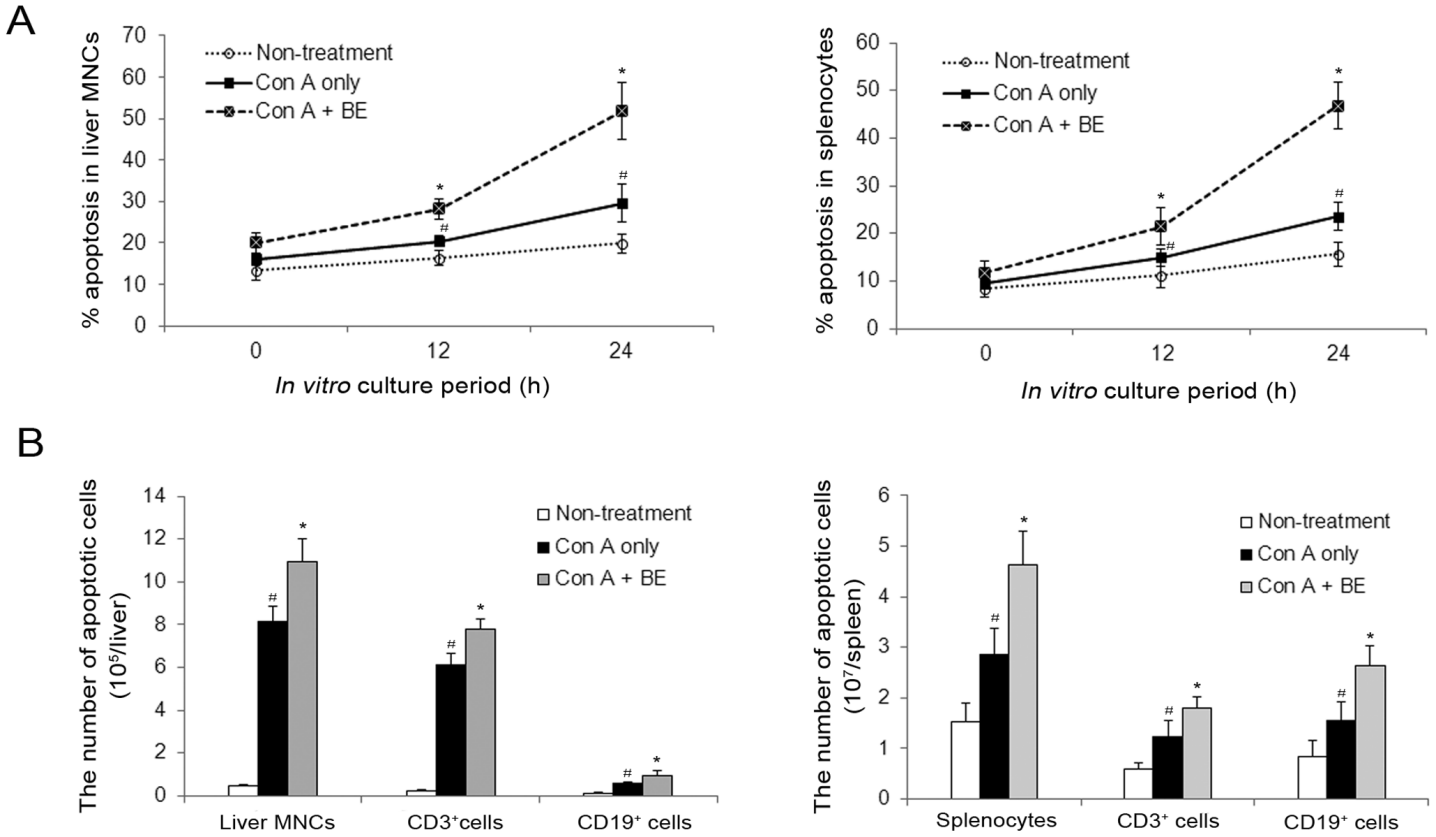
The lymphocytes exposed to BE *in vivo* underwent increased apoptosis upon subsequent culture *in vitro*. Murine hepatitis was induced by an intravenous injection of Con A at a dose of 15 mg/kg. BE (100 mg/kg) was administrated intraperitoneally immediately after Con A injection. The liver MNCs and splenocytes were prepared at 8 h after Con A injection. After 2 hours of in-vitro culture, the adherent macrophages were removed and the nonadherent cells were further cultured *in vitro* for 12 and 24 h. (A) The percentages of apoptosis in the *in vitro* cultured nonadherent cells at each time point were determined by flow cytometry using FITC-annexin V/PI staining. (B) After *in vitro* culture for 24 h, the absolute numbers of apoptotic cells in liver MNCs and splenocytes, as well as in CD3^+^ T cell and CD19^+^ B cell subsets, were determined by cell counting and flow cytometry using PE-anti-CD3/FITC-annexin V staining and PE-anti-CD19/FITC-annexin V staining, respectively. Data are mean ± SEM of 6–8 mice/group. *P<0.05 versus Con A only control, ^#^ *P*<0.05 versus Non-treatment control.

The finding that BE selectively induces apoptosis in activated lymphocytes *in vitro* prompted us to further investigate whether BE could also induce lymphocyte apoptosis *in vivo* and has anti-hepatitis potential using a Con A-induced hepatitis model. As shown in Fig. 5, intravenous administration of Con A resulted in a significant elevation in the serum ALT level at 8 and 24 h after Con A administration. BE treatment significantly reduced the ALT concentrations at both time points (Fig. 5A). Serum levels of IFN-γ and TNF-α were also enhanced in response to Con A challenge after 8 h, which were all suppressed by BE treatment (Fig. 5B). Histologic examination of liver sections showed that Con A challenge resulted in widespread areas of necrosis and massive infiltration of inflammatory cells in livers 24 h after Con A administration. Treatment with BE markedly attenuated these pathologic changes (Fig. 5C). Flow cytometry analysis of hepatic MNCs showed that the total numbers of liver infiltrating MNCs, CD3^+^ T cells, and CD19^+^ B cells were significantly lower in BE-treated mice, compared to mice treated with Con A only, 24 hours after Con A injection (Fig. 5D). Hypothesizing that the reduced numbers of MNCs in the BE treated livers might reflect an increased incidence of apoptosis, we analyzed the incidence of apoptosis among the hepatic MNCs harvested at 24 h after Con A injection with flow cytometry and found that the frequency of apoptosis in liver-infiltrating MNCs significantly increased upon BE treatment (Fig. 5E). Further flow cytometry analysis of freshly isolated splenocytes demonstrated the percentages of apoptotic (Annexin V^+^) cells in splenocytes, splenic CD3^+^ T cells, and CD19^+^ B cell were significantly higher in BE-treated mice than that in mice received Con A only (Fig. 5F and G). However, we noticed that the frequency of apoptosis in liver MNCs only increased from 23.5% to 29.0% upon BE treatment (Fig. 5E), while the total cell numbers of liver MNCs dramatically decreased from 21.8×10^5^ cells per liver decreased to 11.5×10^5^ cells per liver upon BE treatment (Fig. 5D). Thus the absolute number of apoptotic MNCs was calculated to be fewer in BE-treated livers when compared to Con A only control. Nevertheless, it should be noted that, as cells undergo apoptosis, they are recognized and rapidly cleared *in vivo* by phagocytic macrophages [36], [37]. Thus, the flow cytometry data in Fig. 5E did not take into consideration the majority of the cells that had died and had been cleared *in vivo*, but only represented the incidence of apoptosis among liver MNCs that were undergoing apoptosis but had not been cleared at the time point that the cells were harvested (24 h after Con A and BE administration). To overcome the problem of apoptotic cells being cleared *in vivo*, we did *ex vivo* experiments in which we harvested the hepatic MNCs and splenocytes at early stage of BE administration (within 8 h) and cultured them in 24-well flat-bottomed plates for 2 h to allow macrophages adherence, then the nonadherent cells were collected and further cultured for 12 and 24 h to investigate whether these cells would then undergo increased apoptosis *in vitro* in the absence of macrophages. The results showed that during the *in vitro* culture period, the frequency of apoptotic cells raised more readily in the macrophage-depleted live MNCs and splenocytes obtained from mice treated with Con A and BE than in the cells from mice treated with Con A only, so that by 24 h of *in vitro* culture, the increase in the frequency of apoptotic cells in Con A+BE group became more apparent compared with Con A only control (Fig. 6A). At this time point of *in vitro* culture, the absolute numbers of apoptotic cells detected in macrophage-depleted live MNCs and splenocytes, as well as in CD3^+^ T cell and CD19^+^ B cells subsets, were significantly higher in Con A+BE group than in Con A only control (Fig. 6B). These results indicated that lymphocytes exposed to BE *in vivo* undergo increased apoptosis when cultured *in vitro*, suggesting that *in vivo* exposure of lymphocytes to BE makes them more susceptible to apoptosis. These results support the notion that increased levels of apoptosis in lymphocytes is a factor underlying the reduction in MNCs within BE-treated livers. These data together suggested that BE could also promote lymphocyte apoptosis *in vivo* and has anti-hepatitis potential.

## Discussion

In current study, BE is demonstrated to be capable of inducing murine splenocytes to undergo apoptosis with higher efficacy in the presence of Con A than in the absence of Con A. The direct and selective pro-apoptotic effects on activated T and B cells were further corroborated using MACS-purified CD3^+^ splenocytes and CD19^+^ splenocytes. We demonstrated that BE significantly enhanced the apoptosis in pure CD3^+^ splenocytes stimulated with Con A, while only having little effect on naive ones. Consistently, BE was found to be much more effective in inducing Jurkat T cells to undergo apoptosis in the presence of PMA and ionomycin. In pure CD19^+^ splenocytes cultures that had been treated with BE and LPS, the level of apoptosis were greater when compared to that in cultures that treated with BE alone. These results confirm that BE might affect activated lymphocytes to a greater degree than naïve lymphocytes. We also noted that murine BM-derived DCs, murine peritoneal macrophages, and RAW264.7 cells were all insensitive to the BE-induced apoptosis, either activated or inactivated. These results suggested that BE was able to selective promote apoptosis in activated T and B lymphocytes.

Apoptosis is mediated primarily through intrinsic and/or extrinsic pathways [38], [39]. The intrinsic pathway involves altering mitochondrial permeability and subsequent cytochrome c release and formation of the apoptosome, a catalytic multiprotein platform that activates caspase-9. Activated caspase-9 then cleaves caspase-3 resulting in downstream events involved in cell death [38]. The extrinsic pathway is triggered with the ligation of death receptors, followed by the recruitment of an adaptor molecule and procaspase-8 to form a Death-Inducing Signaling Complex, which lead to the autocatalytic cleavage and subsequent activation of caspase-8, and in turn activates caspase-3, leading to apoptosis [39]. Fas is a major death receptor that mediates apoptosis of activated lymphocytes. Upon activation, lymphocytes can express a higher level of Fas and are more susceptible to Fas-mediated apoptosis than naive cells [7]. In this study, we observed that BE did not affects the expressions of Fas and FasL on both T and B lymphocytes, either naïve or activated, but preferentially induced a loss of ΔΨ_m_ in activated lymphocytes. BE treatment also led to the release of cytochrome c from mitochondria to the cytosol, down-regulation of Bcl-2, and up-regulation of Bax in activated lymphocytes. Furthermore, Caspase-9 and caspase-3 but not caspase-8 were activated in activated lymphocytes upon BE treatment. These results indicated that BE induces apoptosis in activated lymphocytes via intrinsic pathway rather than Fas-mediated extrinsic pathway.

BE has been shown to exhibit anti-inflammatory properties on various animal models [21]–[25], but the related mechanisms are only partially understood. Previous studies have associated its anti-inflammatory activities with activation of intestinal Pregnane X Receptor [25], suppression of pro-inflammatory cytokine expression [23], [24], inhibition of nitric oxide production [21], [40], impairment of reactive oxygen intermediates production [20], inhibition of leukocyte adhesion to endothelial cells [20], and modification of eicosanoid synthesis [41]. Data from current study suggest that the anti-inflammatory properties of BE can also be attributed, at least in part, to its ability to trigger apoptosis in activated lymphocytes. This new finding not only extends our understanding on the cellular mechanisms underlying the anti-inflammatory activity of BE, but also raises the possibility that BE may be useful to eliminate activated lymphocytes that contribute to autoimmune diseases.

Con A-induced hepatitis in mice is a well-established mouse model for AIH. In this model, intravenous injection of Con A leads to systemic immune activation, resulting in acute liver injury that resemble the pathology of immune mediated hepatitis in humans [10]. Activated T cells have a critical role in Con A-induced liver damage. Upon Con A challenge, T cells are initially activated and infiltrate the liver tissue, where they induce hepatocyte death either by cell to cell contact or through secretion of pro-inflammatory cytokines, of which TNF-α and IFN-γ are the major ones [42], [43]. In the current study, *in vivo* administration of BE alleviated Con A-induced hepatitis as indicated by decreased transaminase levels and markedly attenuated inflammatory lesions in liver. BE treatment also significantly suppressed Con A-mediated elevation in serum level of TNF-α and IFN-γ, indicating Con A-induced immune activation was inhibited by BE. Furthermore, BE treatment markedly reduced MNCs infiltration in the liver and significantly lowered the absolute number of hepatic T and B lymphocytes. The reduction in hepatic MNCs could possibly result from the decrease in MNCs recruitment into livers and/or the increase in MNCs death and clearance within livers. Since BE can selectively induced apoptosis in activated lymphocytes *in vitro*, we assumed that enhanced apoptosis of lymphocytes may contribute to the reduction of MNCs in livers of BE-treated mice. Indeed, we found that BE treatment increased the incidence of apoptosis among hepatic MNCs and splenocytes in Con A-challenged mice. Flow cytometry assay of annexin V exposure in splenic T and B cells further confirmed that the frequency of splenic T and B lymphocytes undergoing apoptosis was increased in ConA-injected mice upon BE treatment. Furthermore, our *ex vivo* experiments demonstrated that when liver MNCs and splenocytes harvested from mice treated with Con A and BE were cultured *in vitro* for 24 h, both the frequencies of apoptosis and the absolute numbers of apoptotic cells in total liver MNCs and spelnocytes, as well as in CD3^+^ T cell and CD19^+^ B cell subsets, were significantly higher when compared to Con A only control. These *ex vivo* data indicate that lymphocytes from BE-treated mice become increasingly susceptible to apoptosis when cultured *in vitro*. Overall, these results suggest that higher level of apoptosis in lymphocytes is a factor underlying the reduction of MNCs in liver of BE-treated mice, which concur with *in vitro* evidence that BE drives activated lymphocytes to apoptosis. Although we cannot exclude the possibility that BE may execute protective activity through other mechanisms, our data demonstrated that administration of BE enhanced apoptosis of lymphocytes *in vivo*, and pharmaceutical enhancement of lymphocyte apoptosis was therapeutically beneficial in a model of AIH. Nevertheless, the *in vivo* effect of BE was tested here only at one dosage of 100 mg/kg administered by intraperitoneal injection. It would be expected that the oral delivery of BE may have limited utility since the oral bioavailability of BE is very low. Therefore, the present *in vivo* study only shows proof of principle, but does not show any practical utility of the BE in clinic.

Currently the only viable treatments of AIH are immunosuppressant application and liver transplantation [1], [3]. Ideally, the goal of immunosuppressive therapy for autoimmune diseases would be the induction of long-lasting immunological tolerance by selective deleting autoantigen-activated lymphocytes that have failed to undergo apoptosis while leaving the normal immune response to foreign antigens unaffected. Unfortunately, most immunosuppressive drugs (ISDs) currently used for AIH act mainly by preventing the activation and expansion of resting lymphocytes, rather than mediating the elimination of activated lymphocytes [44]–[46]. On the contrary, some ISDs such as cyclosporin A and FK506 even inhibited apoptosis in activated T cells by down-regulation of FasL [47]. Although dexamethasone is known to induce apoptosis in various immune cells, it lack of selectivity for activated lymphocytes [48]. This is why the current ISDs exert global suppressing effects on the immune system, which predispose patients to the development of infection and cancer. Alternatively, selective induction of apoptosis in activated lymphocytes might effectively deplete autoreactive T and B cells without affecting naïve or non-activated ones to avoid intervention of normal immune responses to foreign antigens. In this context, BE would have distinct advantages over the unselective immunosuppressive agents currently used in the treatment of AIH. However, the practical uses of baicalein in pharmaceutical field will depend on the success of attempts to enhance its oral bioavailability. Fortunately, several approaches have been recently developed which could markedly improve the oral bioavailability of BE [49]–[51].

In conclusion, BE selectively promoted apoptosis in activated lymphocytes through intrinsic apoptotic pathway, while having little effect on non-activated ones. Administration of BE alleviates Con A-induced hepatitis in mice and increased the incidences of apoptosis in hepatic MNCs and splenocytes, as well as in CD3^+^ and CD19^+^ splenocytes, in Con A-challenged mice. These observations not only reveal a novel cellular mechanism for the anti-inflammatory effects of BE, but also suggest that it has therapeutic potential for AIH.
