# Correction: Baicalein Selectively Induces Apoptosis in Activated Lymphocytes and Ameliorates Concanavalin A-Induced Hepatitis in Mice

**DOI:** 10.1371/journal.pone.0117635

**Published:** 2015-01-26

**Authors:** The PLOS ONE Staff

The published Fig. 3B includes two panels that are incorrect and duplicate other panels within the same figure. The authors apologize for this error and would like to correct Fig. 3B.

**Figure 3:**
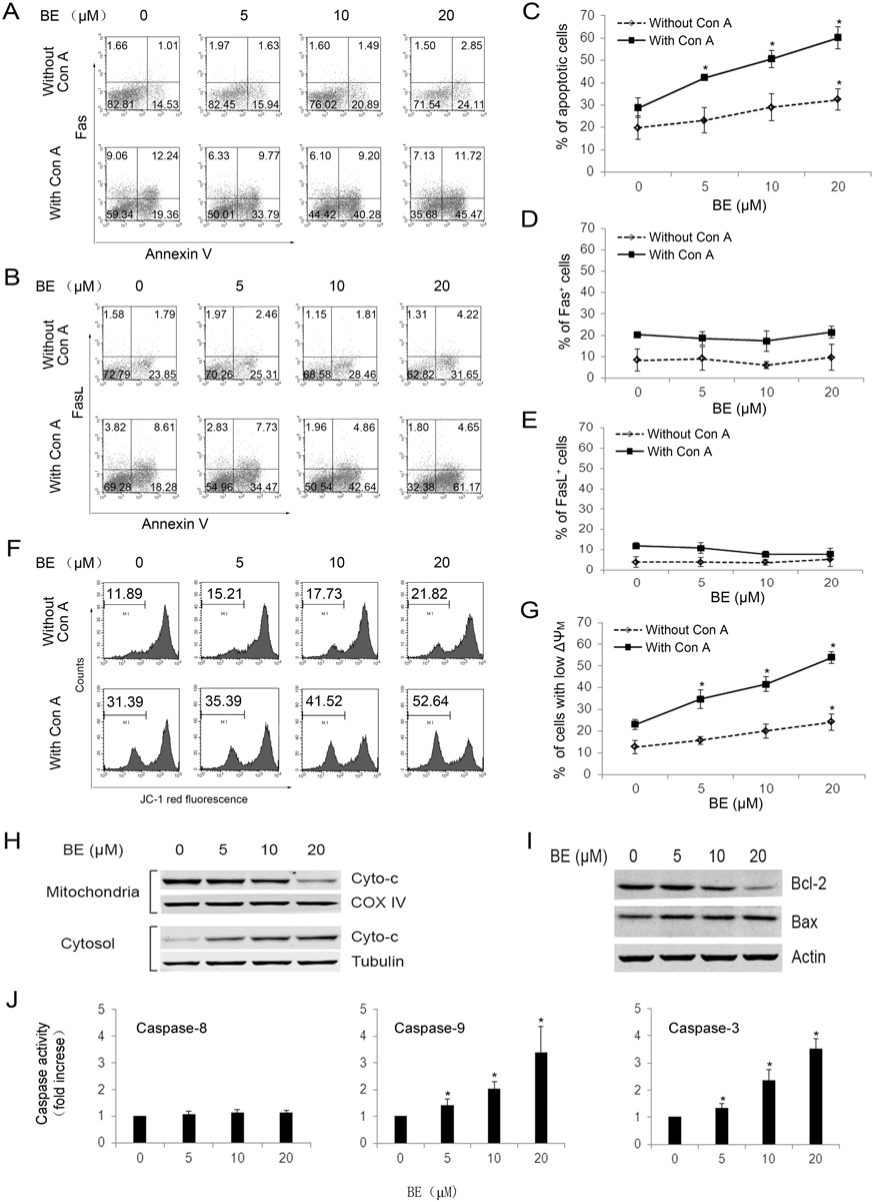
BE selectively induces apoptosis of Con A-activated CD3^+^ T cells through mitochondrial pathway. CD3^+^ T cells were isolated from murine splenocytes using Miltenyi MACS Purification and incubated with indicated concentrations of BE for 24 h in the absence or presence of 5 µg/ml of Con A. (A–E) the percentages of Annexin V^+^, Fas^+^, and FasL^+^ cells were analyzed using PE-anti-Fas mAb/annexin V-FITC or PE-anti-FasL mAb/annexin V-FITC staining. A is a representative of three independent assays with PE-anti-Fas mAb/annexin V-FITC staining. B is a representative of three independent assays with PE-anti-FasL mAb/annexin V-FITC staining. C–E represents mean ± SEM of three independent experiments. (F, G) Loss of ΔΨm in T cells was analyzed using JC-1 staining. F is a representative of three independent assays, and G represents mean ± SEM of three independent experiments. (H) The release of cytochrome c (Cyto-c) from mitochondria in T cells after BE treatment in the presence of Con A was examined by Western blotting. (I) Protein levels of Bcl-2 and Bax in T cells after BE treatment in the presence of Con A were examined by Western blotting. The results shown in H and I are representative of three experiments. (J) The activities of caspase-3, 8, 9 in T cells after BE treatment in the presence of Con A was measured using colorimetric assay. Each column represents the mean ± SEM of 3 experimental values. *P<0.05 versus untreated controls.

The data in Fig. 3B were obtained through flow cytometry analysis of the percentage of FasL-positive cells in both activated T lymphocytes (with Con A stimulation) and naïve T lymphocytes (without Con A stimulation) after BE treatment. During the preparation of the figure, the dot plot obtained for the condition “0 μM BE without Con A” was mistakenly duplicated for the condition of “5 μM BE without Con A”. The dot plot obtained for the condition “5 μM BE with Con A” was duplicated for the condition of “10 μM BE with Con A.” The statistical values in the figure were reported correctly for the corresponding conditions.

We are providing a revised Fig. 3B that displays the correct dot-plots for the conditions of “5 μM BE without Con A” and “10 μM BE with Con A.” The statistical values remain unchanged. These changes do not affect the conclusions reported in the article.
